# Molecular Basis of Wrinkled Variants Isolated From *Pseudoalteromonas lipolytica* Biofilms

**DOI:** 10.3389/fmicb.2022.797197

**Published:** 2022-02-28

**Authors:** Zhenshun Zeng, Shituan Lin, Qian Li, Weiquan Wang, Yuqi Wang, Tangfu Xiao, Yuexue Guo

**Affiliations:** ^1^Key Laboratory for Water Quality and Conservation of the Pearl River Delta, Ministry of Education, School of Environmental Science and Engineering, Guangzhou University, Guangzhou, China; ^2^Key Laboratory of Tropical Marine Bio-Resources and Ecology, Guangdong Key Laboratory of Marine Materia Medica, RNAM Center for Marine Microbiology, South China Sea Institute of Oceanology, Chinese Academy of Sciences, Guangzhou, China; ^3^University of Chinese Academy of Sciences, Beijing, China; ^4^State Key Laboratory of Geohazard Prevention and Geoenvironment Protection, Chengdu University of Technology, Chengdu, China

**Keywords:** *Pseudoalteromonas*, biofilm, genetic variation, wrinkled variant, cellulose production

## Abstract

Many *Pseudoalteromonas* species are dominant biofilm-forming Gammaproteobacteria in the ocean. The formation of *Pseudoalteromonas* biofilms is often accompanied by the occurrence of variants with different colony morphologies that may exhibit increased marine antifouling or anticorrosion activities. However, the genetic basis of the occurrence of these variants remains largely unexplored. In this study, we identified that wrinkled variants of *P. lipolytica* mainly arose due to mutations in the *AT00_08765*, a *wspF-like* gene, that are associated with decreased swimming motility and increased cellulose production. Moreover, we found that the spontaneous mutation in *flhA*, encoding a flagellar biosynthesis protein, also caused a wrinkled colony morphology that is associated with cellulose overproduction, indicating that *flhA* plays a dual role in controlling flagellar assembly and polysaccharide production in *P. lipolytica*. Investigation of wrinkled variants harboring spontaneous mutation in *dgcB*, encoding a GGDEF domain protein, also demonstrated *dgcB* plays an important role in regulating cellulose production and swimming motility. In addition, by screening the suppressor of the *AT00_08765* variant strain, we also identified that the spontaneous mutation in *cheR* and *bcsC* directly abolished the wrinkled phenotype of the *AT00_08765* variant strain, suggesting that the chemosensory signaling transduction and cellulose production are crucial for the determination of the wrinkled phenotype in *P. lipolytica*. Taken together, this study provides insights into the genetic variation within biofilms of *P. lipolytica*.

## Introduction

Many bacterial species are capable of living in matrix-encased structures known as biofilms. During the development of biofilms, bacteria express phenotypic traits that are often distinct from those that are expressed during planktonic growth (Flemming and Wuertz, 2019). Importantly, self-generated diversity often occurs through mutation or recombination following the biofilm mode of growth due to evolution by adapting to the heterogeneous spatial structure within biofilms (Boles et al., 2004; Stewart and Franklin, 2008). Investigation of the genetic variation within biofilms would provide valuable information for studying bacterial evolution and adaptation, which would help deepen our understanding of pathogen infection, ecological processes, and other processes mediated by microorganisms (Matz et al., 2005; Hansen et al., 2007; Schick and Kassen, 2018).

The exploration of biofilm mediated genetic diversity has particularly focused on some pathogenic bacteria with a strong biofilm formation ability, such as *Pseudomonas aeruginosa* (Harris et al., 2021), *Burkholderia cenocepacia* (Traverse et al., 2013) and *Acinetobacter baumannii* (Santos-Lopez et al., 2019). Pathogenic bacteria residing on tissues or indwelling devices that experience genetic variation often result in elevated tolerance to antimicrobials and the host immune response, and reduced swimming motility on semisolid agar plates (Winstanley et al., 2016; Pestrak et al., 2018). Generally, the evolved genetic variants in biofilms were detected primarily as changes in colony morphology, such as smooth and medium colony to small (Wang et al., 2014), mucoid (McElroy et al., 2014; Schwartbeck et al., 2016), translucent (Kaluskar et al., 2015; Tipton et al., 2015) and wrinkled (or rugose) colony morphologies (Ali et al., 2002; Grau et al., 2005; Dragos et al., 2017). Among them, wrinkled colony morphology variants dramatically differ in biofilm formation abilities and expression of biofilm-related genes, which is crucial for the process of pathogenesis and fitness improvement (Gloag et al., 2019; Chavez-Dozal et al., 2021).

Genetic analysis revealed that mutations in various genes were responsible for the occurrence of wrinkled phenotypes in different bacteria. For example, a spontaneous mutation in the *wspF* gene, a *cheB-like* gene that encodes a methylesterase in the chemosensory system, was commonly found in both early laboratory-grown biofilms and clinical isolates of *P. aeruginosa* (Porter et al., 2011). Mutations in *wspF* result in an elevated concentration of c-di-GMP, a ubiquitous second messenger that regulates cell surface-associated traits in bacteria and thus increases the production of Pel and Psl polysaccharides, leading to a wrinkled colony morphology (Hickman et al., 2005; McElroy et al., 2014; Gloag et al., 2019). In addition, the wrinkled colony morphology is also associated with mutations in many other genes that increase extracellular polysaccharide production in *P. aeruginosa*, such as *fleQ*, encodes a c-di-GMP binding factor (Hickman and Harwood, 2008); *bifA*, encodes a c-di-GMP phosphodiesterase (Kuchma et al., 2007); *armZ*, encodes a diguanylate cyclase repressor (Jones et al., 2014); and *rsmA*, encodes a posttranscriptional regulator target psl mRNA (Irie et al., 2010). Moreover, a molecular investigation of the biofilm variant of *Pseudomonas fluorescens* also linked the occurrence of wrinkly colony morphology to the spontaneous mutation of *wspF*, *awsX* or *mwsR*, which deregulate diguanylate cyclases, leading to the overproduction of c-di-GMP and the stimulation of cellulose expression (McDonald et al., 2009; Lind et al., 2017). Besides, a null mutation in *hapR*, a master biofilm transcriptional regulator, also promoted the development of a rugose colony morphology in *Vibrio cholera* EI Tor, but not in classical biotypes of *V. cholera*, which is associated with increased production of *Vibrio* polysaccharide (VPS*^ETr^*; Jobling and Holmes, 1997; Yildiz et al., 2004). Recently, it was proved that the expression of BipA, a ribosome-associated GTPase, is critical for the HapR mediated rugosity in both solid–air (i.e., colony morphology) and air–liquid (i.e., pellicles) interfaces (Del Peso Santos et al., 2021). Additionally, phase variation between smooth and rugose phenotypes also can be controlled by a gain of function point mutation in the diguanylate cyclase (VpvC), which act independently from the role of HapR in *V. cholera* (Beyhan and Yildiz, 2007).

Moreover, it has also been demonstrated that deletion of genes encoding the flagellar regulators (*flrA*, *flrB*, *flrC*, and *fliA*), components of the C-ring (*fliG*, *fliM*, and *fliN*), the flagellar T3SS (*flhA*, *flhB*, *fliI*, *fliH*, and *fliJ*), the flagellar hook (*flgE*), and the capping protein (*fliD*), leading to an incomplete flagellum biogenesis, promoted wrinkled colony morphology in *V. choleare* due to trigger c-di-GMP signaling pathways that increase VPS polysaccharide production (Wu et al., 2020). Similar work on *P. aeruginosa* that looked in depth into the relationship between flagellar biogenesis, c-di-GMP accumulation, and EPS production was also revealed that flagellar mutants were selected for at high frequencies in biofilms, which activated Pel and Psl overproduction and thus developed the rugose small colony variants (Harrison et al., 2020).

*Pseudoalteromonas* is an important bacterial genus that is commonly found in association with abiotic and biotic surfaces in marine environments (Holmstrom and Kjelleberg, 1999; Bowman, 2007). In our previous study, we demonstrated that the marine non-pathogenic bacterium *Pseudoalteromonas lipolytica* also produced distinctly wrinkled colony morphology variants during biofilm formation that exhibited a high marine antifouling and steel anticorrosion activity compared to the ability of the wild-type strain to exhibit smooth colony morphology (Zeng et al., 2015; Liu et al., 2018). However, the molecular basis of the wrinkled colony morphology variants of *P. lipolytica* remains largely unexplored. We previously reported that a point mutation in *AT00_08765* (or *RS08395*), a *wspF-like* gene, which product is a methylesterase that shares a domain architecture similar to the chemotaxis protein CheB, causes the wrinkled colony morphology of *P. lipolytica* due to cellulose overproduction, but there are also other wrinkled variants in which no mutation in the *AT00_08765* gene was identified (Zeng et al., 2015). In this study, we aimed to further identify the spontaneously mutated genes that cause the alteration of smooth colony morphology to wrinkled colony morphology in *P. lipolytica* through whole-genome resequencing combined with genetic manipulation. Moreover, swimming motility and polysaccharide production were also investigated in these wrinkled variants by comparison with the wild-type strain.

## Materials and Methods

### Strains and Growth Conditions

The bacterial strains used in this study are listed in Supplementary Table 1. All bacterial strains were stored at −80°C, and the frozen stocks were streaked onto fresh agar plates before experiments were performed. *Escherichia coli* WM3064 were grown in LB supplemented with 0.3 mM DAP (2,6-diamino-pimelic acid) at 37°C. *Pseudoalteromonas lipolytica* strains were grown in SWLB (seawater LB, 1% tryptone and 0.5% yeast extract dissolved in seawater) or 2216E at 25°C. Kanamycin (50 μg/mL) and erythromycin (25 μg/mL) were used to maintain the gene knockout vector pK18mob*sacB*-ery in *E. coli* and *P. lipolytica*, respectively. Chloramphenicol (30 μg/mL) was used to maintain the expression vector pBBR1MCS-cm in both *E. coli* and *P. lipolytica*.

### Isolation of Biofilm Variants

The isolation of colony morphology variants from biofilms was conducted as we previously described (Zeng et al., 2015). Briefly, pellicle biofilms of *P. lipolytica* wild-type and EPS+ strains were grown in SW-LB medium in test tubes without shaking for the indicated times at 25°C. The harvested biofilm cells were then diluted in seawater using 10-fold serial dilutions and subsequently plated on fresh SWLB agar plates to obtain 30–300 colonies per plate. The wrinkled or smooth colonies were examined after 3 days of incubation at 25°C. The randomly selected variant strains were inoculated into fresh liquid medium or onto agar plates for three rounds of overnight passaging to ensure the stable variation. The *AT00_08765* gene was sequenced in 24 wrinkled variants using the sequencing primers listed in Supplementary Table 2.

### Motility Assay

A swimming motility assay was conducted on semisolid agar plates. Overnight cultures of the indicated strains were inoculated onto 2216E plates containing 0.25% agar (w/v) and incubated for the indicated times at room temperature. The diameter of the swimming motility was also measured after incubation. The assays were performed with three independent colonies for each strain.

### Congo Red Binding Assay

Congo red (CR) binding assay was conducted on agar plates or liquid cultures containing different concentrations of CR. Bacterial strains producing cellulose lead to the pink or red phenotype on Congo red agar plates. Briefly, fresh colonies were streaked onto 2216E agar plates containing 100 μg/mL CR and incubated at 25°C for 3 days. An increasing depth of color indicated high cellulose matrix production levels. To quantify cellulose production, 20 μg/mL CR was added to 1 ml of overnight culture and continued to culture with shaking condition for 3 h. The cultures were then centrifuged at 15,000 rpm for 5 min. The cell pellet was examined and the supernatant was collected. The amount of CR bound to the cells was determined by measuring the absorbance of the supernatant at 490 nm as previously described (Sajadi et al., 2019). Each sample was prepared and assayed in triplicate.

### Transmission Electron Microscopy

*Pseudoalteromonas lipolytica*Δ*flhA* mutant and the EPS+ strains were grown on 2216E agar medium at 25°C. The fresh grown colonies were inoculated in 2216E medium with an adjustment OD_600_ between 0.5 and 1.0. The bacterial suspension was transferred to a formvar-coated copper mesh membrane for 2 min and then covered with 30 g/l phosphotungstic acid at pH 7.0 for another 2 min. After air drying the membrane, the cells were observed and imaged using a Hitachi H-7650 microscope.

### Construction of Gene Deletion Mutants and Expression Plasmids

Gene deletion mutants were constructed using our previously published method (Wang et al., 2015). Primers used in this study are listed in Supplementary Table 2. Briefly, the upstream and downstream regions of the target gene open reading frame were PCR-amplified from wild-type genomic DNA. The two fragments were then ligated into the suicide plasmid pk18mob*sacB*-ery to create the deletion vector in a host of *E. coli* WM3064, and then integrated into the chromosome of the *P. lipolytica* strain by conjugation. The *P. lipolytica* deletion mutants were generated by screening for erythromycin resistance followed by *sacB*-based counterselection. The vector pBBR1MCS-Cm was used for gene expression in *P. lipolytica*. The target gene with its native promoter was PCR amplified and ligated into the vector pBBR1MCS-Cm in a host of *E. coli* WM3064. The plasmid was transferred to *P. lipolytica* by conjugation as described above. The resulting deletion mutants and expression plasmids were further confirmed by sequencing using the primer sets pK18-f/pK18-r and pBBR1MCS-f/pBBR1MCS-r, respectively, listed in Supplementary Table 2.

### Whole-Genome Re-sequencing

The genomes of four biofilm variant strains (V3, V4, SV1 and SV2) were sequenced using the whole-genome shotgun method by GENEWIZ Co., Ltd. (Suzhou, Jiangsu, China). The raw sequencing data were processed after filtering, and the statistics of the sequencing data are listed in Supplementary Table 3. In total, the average clean sequencing data for each variant strain was greater than 3000 Mb, and the average genome sequencing depth exceeded 500. SNPs and InDels were detected based on the aligned result of the assembly sequence and the *P. lipolytica* reference. The mutation results revealed by whole-genome re-sequencing are listed in Tables 2, 3. Mutations in *flhA*, *dgcB wspC* and *bcsC* in the corresponding variant strains were also further confirmed by PCR-amplification and sequenced using the primer sets listed in Supplementary Table 2. The whole-genome sequencing project has been submitted to the SRA database under the accession numbers PRJNA756106, PRJNA756166, PRJNA755839 and PRJNA755904 for V3, V4, SV1, and SV2, respectively.

## Results

### The Wrinkled Variant Reduced Swimming Motility While Increasing Cellulose Production

**FIGURE 1:**
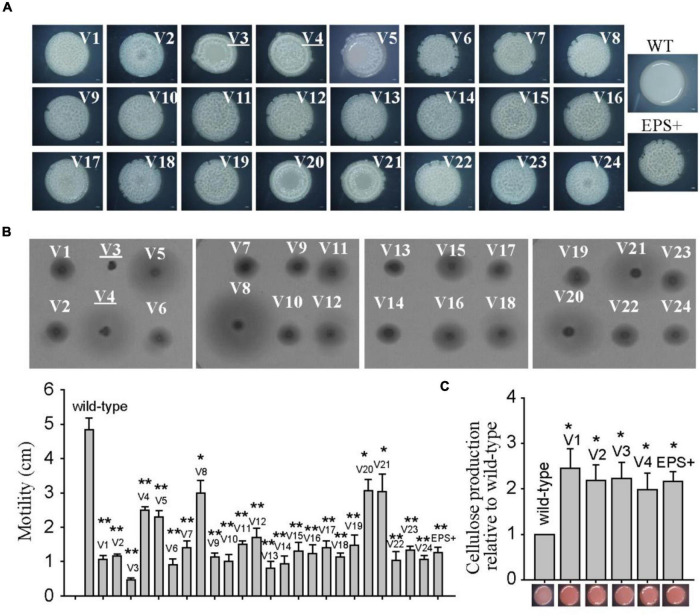
The wrinkled variant reduced swimming motility while increasing cellulose production. **(A)** The colony morphologies of twenty-four wrinkled variants were examined by stereoscopic microscopy after 3 days of incubation on SWLB agar plates. The colony morphologies of wild-type *P. lipolytica* and the EPS+ strain, a wrinkled variant, are also presented. **(B)** Swimming motility of the wrinkled variants was examined and measured by culturing on semisolid 2216E agar plates for 20 h. **(C)** Cellulose production was examined and measured using Congo red for the wild-type and different wrinkled variants (representative V1 to V4 and EPS+ strains were shown). The data are from three independent cultures. Standard deviations are shown, and statistical significance (*P < 0.05; **P < 0.01; NS, not significant) is indicated with asterisks. Images shown in Figures 1–5 are representative images.

Previously, we found that *Pseudoalteromonas lipolytica* produced a number of wrinkled colony morphology variants during the development of biofilms (Zeng et al., 2015). In this study, we further characterized 24 wrinkled variant strains that were randomly isolated from *P. lipolytica* biofilms. As shown in Figure 1A, all 24 isolated variant strains, designated V1 to V24, exhibited wrinkled colony morphology, which shared a similar phenotype to the EPS+ wrinkled variant, as we have previously reported (Liu et al., 2018). Swimming motility assays showed that all of the variant strains dramatically decreased swimming motility by approximately 2- to 10-fold compared to that of the wild-type strain after 20 h of incubation (Figure 1B). Among them, the V3 strain was completely defective in swimming motility on semisolid agar plates, whereas V4, V5, V8, V20, and V21 exhibited approximately twofold reduced swimming motility compared to that of the wild-type strain. In addition, the wrinkled variant strains showed a darker red color on Congo red plates than the wild-type strain, suggesting that the variant strains produced more cellulose polysaccharide than the wild-type strain (Figure 1C and Supplementary Figure 1). As shown in Figure 1C, the cellulose production of the variant strains increased approximately twofold to threefold compared to that of the wild-type strain. Thus, the self-generated wrinkled colony morphology variant strains in *P. lipolytica* biofilms showed reduced swimming motility and increased cellulose production.

### Spontaneous Mutation in the *AT00_08765* Gene Cause the Wrinkled Phenotype in the Majority of Wrinkled Variants

**FIGURE 2:**
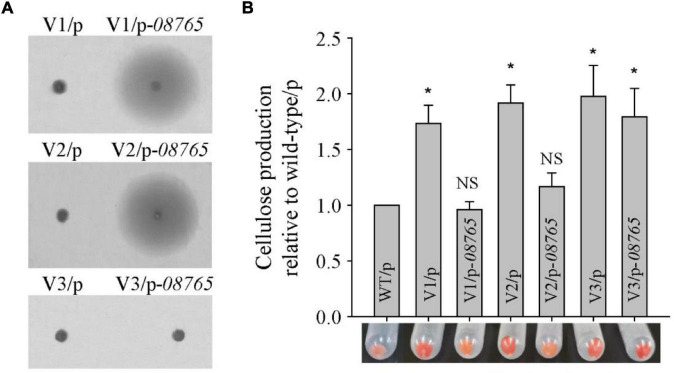
Spontaneous mutation in the *wspF-like* gene causes the wrinkled phenotype in the majority of wrinkled variants. **(A)** Swimming motility of three wrinkled variants complementing the *wspF-like* gene was examined by culturing on semisolid 2216E agar plates containing 15 μg/mL chloramphenicol for 20 h. **(B)** Cellulose production was examined and measured using Congo red for three wrinkled variants complemented with the *wspF-like* gene. Statistical significance (*P < 0.05; NS, not significant) is indicated with asterisks.

We previously identified that the colony morphology of the wrinkled variant was associated with mutation in the *AT00_08765* gene (Zeng et al., 2015). Thus, the total genomic DNA of these twenty-four wrinkled colony morphology variant strains was extracted and used as templates to amplify the *AT00_08765* genes for sequencing. The results showed that nineteen variant strains harbored mutations at different positions within the *AT00_08765* gene, except for the V3, V4, V5, V20, and V21 variant strains (Table 1). The mutation types include base deletion, base insertion or base substitution, which result in frameshift mutation, nonsense mutation or non-synonymous mutation in the *AT00_08765* gene, leading to a defective gene product. To further confirm the physiological function of AT00_08765, the *AT00_08765* gene from the wild-type strain was cloned *via* the broad-host-range vector pBBR1MCS and then expressed in three wrinkled variant strains (V1 to V3). The results showed that expression of the wild-type AT00_08765 significantly restored swimming motility for the variant V1 and V2 strains that harbored mutation in *AT00_08765*, but not for the V3 variant strain that did not harbor mutation in *AT00_08765* (Figure 2A). Moreover, the expression of AT00_08765 also restored cellulose production to the wild-type level for the V1 and V2 strains, whereas cellulose production for the V3 variant strain remained unchanged (Figure 2B). Taken together, we demonstrated that the phenotypes of the majority of wrinkled variants were mainly caused by spontaneous mutations in the *AT00_08765* gene.

**TABLE 1:** Mutations in *wspF* in 19 wrinkled variant strains.

Number	Mutation type	Nucleotide position	Nucleotides change	Consequence
V1	Deletion	1024	A deleted	Frame shift
V2	SNP	1012	C to A	Gln to Lys
V6	SNP	720	T to A	Non-sense
V7	Deletion	1008	TTGG inserted	Frame shift
V8	Deletion	959–966	ATGGTCGT deleted	Frame shift
V9	SNP	875	C to T	Pro to Leu
V10	SNP	875	C to T	Pro to Leu
V11	Deletion	1061–1105	45 bp deleted	Frame shift
V12	SNP	875	C to T	Pro to Leu
V13	SNP	974	C to A	Ala to Asp
V14	Deletion	950	G deleted	Frame shift
V15	Deletion	1141	G deleted	Frame shift
V16	Deletion	1061–1105	45 bp deleted	Frame shift
V17	SNP	1025	C to A	Thr to Asn
V18	SNP	1025	C to A	Thr to Asn
V19	SNP	974	C to A	Ala to Asp
V22	Insertion	1008	TTGG inserted	Frame shift
V23	Deletion	731	T deleted	Frame shift
V24	SNP	1212	C to A	Gln to Lys

**TABLE 2:** Mutations revealed by whole-genome re-sequencing of the two wrinkled variant strains isolated from the biofilms of wild-type *P. lipolytica*.

Number	Gene	Product	Position	WT	V3	V4	Consequence
M1	RS00455	Unknown	100710	A	T	T	Non-syn
M2	RS01850	Cryptochrome DASH	423205	G	T	T	Non-syn
M3	RS02830	Unknown	659933	C	T	T	Non-syn
M4	RS09230	tonB-dependent receptor	607054	T	C	T	Non-syn
M5	Intergenic		1063413	T	A	A	
M6	RS12195	GGDEF DgcB	112740	T	T	G	Non-syn
M7	RS13615	Unknown	444267	T	A	A	Syn
M8	RS13870	Unknown	500981	G	A	A	Non-syn
M9	RS14650	Glyoxalase family	689010	G	T	T	Syn
M10	RS15960	Peptidase S9	97104	A	T	T	Non-syn
M11	RS16600	Unknown	262969	G	A	A	Syn
M12	RS18860	Transposase	103954	A	G	G	Non-syn
M13	RS19020	Oxidoreductase	134686	T	G	G	Syn
M14	RS19145	EAL and GGDEF	159082	G	T	T	Syn
M15	RS08430	Flagellar biosynthesis FlhA	407227	T	T inserted	T	Frameshift
M16	RS13655	Unknown	450256	T	108 bp inserted	T	Frameshift

**TABLE 3:** Mutations revealed by whole-genome re-sequencing of the two smooth colony variant strains isolated from the biofilms of *P. lipolytica* EPS+.

Number	Gene	Product	Position	WT	EPS+	SV1	SV2	Consequence
M1	RS08395	Methylesterase CheB	398626	T	A	A	A	Non-sense
M2	RS08640	Methyltransferase CheR	451429	A	A	C	A	Non-syn
M3	RS16820	Efflux RND transporter	319379	C	C	T	C	Non-syn
M4	RS17835	PH domain-containing protein	539046	G	G	G	T	Non-sense
M5	RS18285	DNA topoisomerase subunit GyrB	639790	G	G	G	A	Syn
M6	RS18420	Hypothetical protein	669175	T	T	T	G	Non-syn
M7	RS02190	Cellulose synthase subunit BcsC	510497	A	A	A	–	Frameshift
M8	RS14825	Lipoprotein	728093–728102	AGTTTTGGCA	AGTTTTGGCA	–	–	Frameshift

### Spontaneous Mutation in *flhA* Causes the Phenotypes of the V3 Wrinkled Variant

**FIGURE 3:**
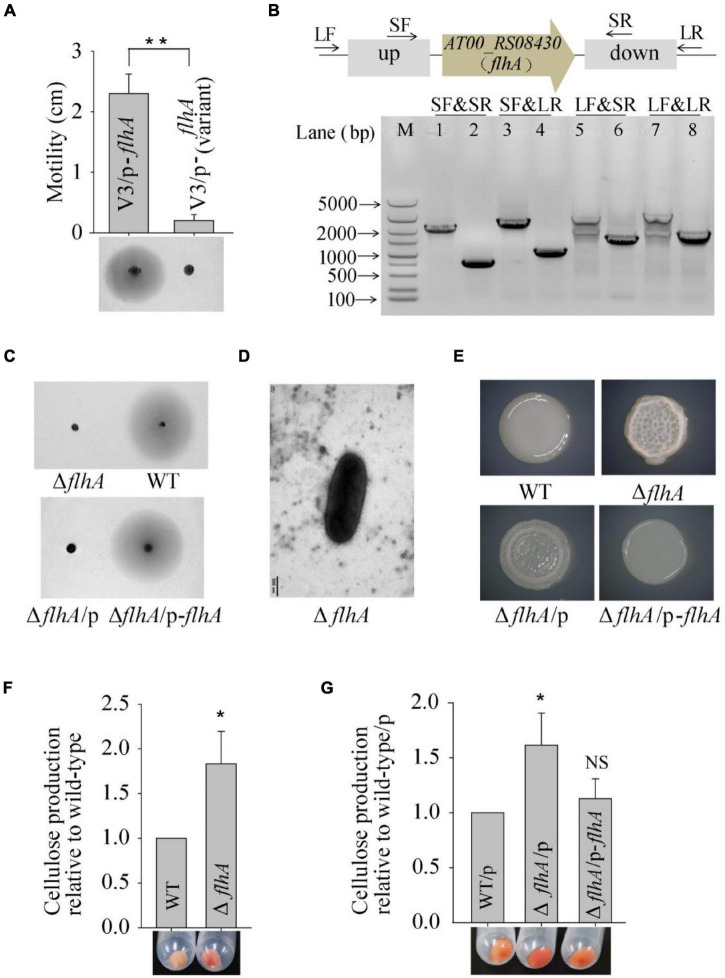
Spontaneous mutation in *flhA* causes the phenotype of the V3 wrinkled variant. **(A)** Swimming motility of the V3 variants complementing the *flhA* gene was examined by culturing on semisolid 2216E agar plates containing 15 μg/mL chloramphenicol for 20 h. **(B)** In-frame deletion of *flhA* in *P. lipolytica* wild-type strain was confirmed by PCR using four primer sets (SF&SR, SF&LR, LF&SR, and LF&LR) flanking the open reading frame of *flhA* (up panel). M indicated marker. Lanes 1, 3, 5 and 7 used DNA from the wild-type strain, and lanes 2, 4, 6 and 8 used DNA from the *flhA* mutant strain. **(C)** Swimming motility of the wild-type and Δ*flhA* mutant strain and its complementation strain. **(D)** Morphology of the Δ*flhA* mutant strain examined by transmission electron microscopy. **(E)** The colony morphologies of the wild-type and Δ*flhA* mutant strain and its complementation strains. **(F)** Cellulose production by the wild-type and Δ*flhA* mutant strain. **(G)** Cellulose production by the Δ*flhA* mutant strain complementing the *flhA* gene. Statistical significance (*P < 0.05; **P < 0.01; NS, not significant) is indicated with asterisks.

To explore the molecular basis of the wrinkled variant without harboring a mutation in the *AT00_08765* gene, we performed a whole-genome re-sequencing approach based on the genome sequence of wild-type *P. lipolytica* to identify the genomic change in the V3 and V4 wrinkled variants, which exhibited relatively lower and higher activity in swimming motility, respectively, compared to other wrinkled variants (Figure 1). Statistically, both of the genomes of the V3 and V4 strains showed 99.83% coverage to the reference wild-type genome, and the sequencing depths both exceeded 500× (Supplementary Table 3). By aligning the sequences to that of the reference wild-type genome, sixteen sites of mutations (designated M1 to M16) were found in these two variants. Of these sixteen mutations, fourteen were point mutations, including five synonymous mutations and eight non-synonymous mutations located in the coding region, and one mutation located in the intergenic region. Besides, two insertion mutations were also found located in the coding region (Table 2). Given that the V3 and V4 variants exhibited different swimming motility activities, the mutated genes that determine the alteration of swimming motility should be different from each other. Thus, three mutations of M4, M15, and M16 that were only found in the V3 variant strain and one mutation of M6 that was only found in the V4 variant strain might be critical in determining the corresponding phenotype for the V3 and V4 variants. Among them, M15 was located at *AT00_RS08430*, which encodes a flagellar biosynthesis protein FlhA that is required for formation of the rod structure of the flagellar type III protein export apparatus (Terahara et al., 2018). To verify that *flhA* mutation in *P. lipolytica* causes defects in swimming motility, the intact *flhA* gene from the wild-type strain and the variant *flhA* gene from the V3 variant strain were each cloned *via* the broad-host-range vector pBBR1MCS, and then expressed in the V3 variant strain, respectively. The results showed that the expression of wild-type *flhA* to the V3 variant successfully restored swimming motility, whereas the expression of the variant *flhA* failed to do so (Figure 3A). To further investigate the physiological function of FlhA, an in-frame deletion of the *flhA* gene was constructed in *P. lipolytica* (Figure 3B). As expected, the deletion mutant strain Δ*flhA* completely lost swimming motility as the V3 variant strain, and complementation of *flhA* to the Δ*flhA* mutant strain also restored swimming motility (Figure 3C). To verify FlhA is required for the flagellar assembly, we examined the cell morphology of the Δ*flhA* mutant strain using transmission electron microscopy. As expected, *flhA* mutation causes defects in flagellar assembly (Figure 3D). In contrast, *P. lipolytica* wild-type strain harbor a single polar flagellar (Zeng et al., 2015). Moreover, the Δ*flhA* mutant strain displayed wrinkled colony morphology on SW-LB agar medium, and complementation of *flhA* to the Δ*flhA* mutant strain also restored smooth colony morphology, which was similar to that of the wild-type strain (Figure 3E). Importantly, the Δ*flhA* mutant strain also increased cellulose production approximately twofold, which is similar to that noted for the V3 variant strain (Figure 3F), whereas the expression of *flhA* in the Δ*flhA* mutant strain reduced cellulose production to the wild-type level (Figure 3G). Taken together, these results demonstrated that the wrinkled variant of *P. lipolytica* harboring a mutation in *flhA* gene cause defects in swimming motility and an overproduction of cellulose.

### The V4 Wrinkled Variant Harbors a *dgcB* Mutation That Increases Swimming Motility

**FIGURE 4:**
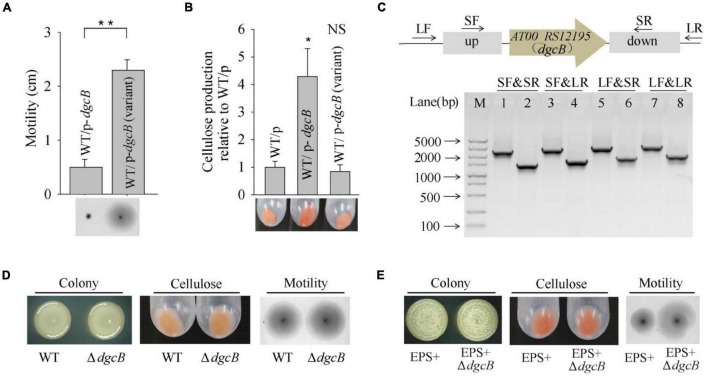
The V4 wrinkled variant harbors a *dgcB* mutation that increases swimming motility. **(A)** Swimming motility of the wild-type strain expressing DgcB from the wild-type and V4 variant strains. **(B)** Cellulose production by the wild-type strain expressing DgcB from the wild-type and V4 variant strains. **(C)** In-frame deletion of *dgcB* in *P. lipolytica* wild-type strain was confirmed by PCR using four primer sets flanking the open reading frame of *dgcB* (up panel). M indicated marker. Lanes 1, 3, 5 and 7 used DNA from the wild-type strain, and lanes 2, 4, 6 and 8 used DNA from the *dgcB* mutant strain. **(D)** The colony morphology, cellulose production and swimming motility of the wild-type and Δ*dgcB* mutant strains. **(E)** The colony morphology, cellulose production and swimming motility of the EPS+ and EPS+ Δ*dgcB* mutant strains. Statistical significance (*P < 0.05; **P < 0.01; NS, not significant) is indicated with asterisks.

Based on the whole-genome re-sequencing results, the M6 mutation might cause phenotypic changes in the V4 variant strain. M6 was located at *AT00_RS12195*, which encodes a diguanylate cyclase DgcB that mediates the synthesis of a c-di-GMP molecule. To investigate the physiological function of the variant *dgcB*, the *dgcB* gene from the wild-type strain and from the V4 variant strain were each cloned and expressed in the wild-type strain. The results showed that overproduction of wild-type DgcB dramatically decreased swimming motility while increasing cellulose production; however, overproduction of the variant DgcB from the V4 variant failed to do so (Figures 4A,B). To further test the physiological function of wild-type DgcB, an in-frame deletion of the *dgcB* gene was constructed in *P. lipolytica* (Figure 4C). However, the results showed that the colony morphology, swimming motility and cellulose production of the Δ*dgcB* mutant strain displayed similar characteristics to those of the wild-type strain (Figure 4D). More than forty genes encode a diguanylate cyclase in the genome of *P. lipolytica*, and twelve of them were annotated as *dgcB* (data not shown). This result indicates that deletion of one *dgcB* gene (*AT00_RS12195*) in wild-type *P. lipolytica* might have a minor impact on physiological performance. We next sequenced the *AT00_RS12195* gene in other biofilm wrinkled variants that have a higher swimming motility as shown Figure 1B. The results showed that biofilm variants V20 and V21 also harbor the same point mutation in *AT00_RS12195* (data not shown). Thus, the increasing swimming motility of these wrinkled variants might be correlated with the *AT00_RS12195* mutation. Therefore, we deleted *AT00_RS12195* in the *P. lipolytica* EPS+ host, a wrinkled variant, to investigate the corresponding phenotype. Although deletion of *AT00_RS12195* in the EPS+ host induced minimal effects on the colony morphology and cellulose production level, the EPS+ Δ*AT00_RS12195* strain displayed higher swimming motility than the EPS+ variant strain after 20 h of incubation on swimming agar medium (Figure 4E). Taken together, these results showed that the *AT00_RS12195* mutation increased swimming motility in wrinkled variants of *P. lipolytica*, and overproduction of AT00_RS12195 led to cellulose overproduction and decreased swimming motility.

### Spontaneous Mutations in *cheR* or *bcsC* Suppress the Wrinkled Colony Morphology of the *Pseudoalteromonas lipolytica* EPS+ Strain

**FIGURE 5:**
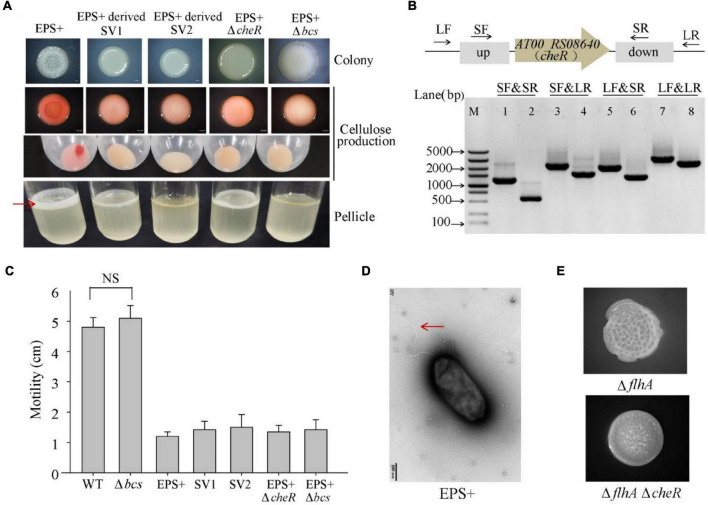
Spontaneous mutations in *cheR* or *bcsC* suppress the wrinkled colony morphology of the *P. lipolytica* EPS+ strain. **(A)** The colony morphologies, cellulose production, and pellicle formation of the *P. lipolytica* EPS+ strain, two suppressor strains and two gene deletion mutant strains of the EPS+ variant. Red arrow indicates bacterial pellicle formed in air-liquid interface after 5 days of incubation. **(B)** In-frame deletion of *cheR* in *P. lipolytica* EPS+ was confirmed by PCR using four primer sets flanking the open reading frame of *cheR* (up panel). M indicated marker. Lanes 1, 3, 5 and 7 used DNA from the wild-type strain, and lanes 2, 4, 6 and 8 used DNA from the *cheR* mutant strain. **(C)** Swimming motility of the indicated strains after 24 h of incubation. **(D)** Morphology of the *P. lipolytica* EPS+ strain examined by transmission electron microscopy. Arrow indicates flagellar. **(E)** The colony morphologies of the Δ*flhA* and Δ*flhA*Δ*cheR* double deletion mutant strains.

To further identify the genetic factor associated with the wrinkled colony morphology, we screened suppressor variant strains that switched the phenotype from wrinkled to smooth within a 5-day-old biofilm of *P. lipolytica* EPS+, a wrinkled variant strain with an *AT00_08765* gene mutation. As shown in Figure 5A, two suppressor variant strains with stable smooth colony morphology, designated SV1 and SV2, were isolated from the ancestral strain EPS+. We next performed a whole-genome re-sequencing approach based on the genome sequence of the *P. lipolytica* EPS+ strain to identify the genomic change that occurred in these two variants. Statistically, both of the genomes of SV1 and SV2 showed 99.83% coverage compared to the genome of the EPS+ strain, accompanied by a sequencing depth greater than 500× (Supplementary Table 3). By aligning the genome sequences to that of the reference genome, eight sites of mutations were found in these two variants, in which the point mutation in *AT00_08765* originally existed in the EPS+ strain was also listed in Table 3. Notably, the genome of SV1 harbors a non-synonymous mutation in *AT00_09010* (or *AT00_RS08640*), which encodes a methyltransferase CheR that mediates chemosensory signaling in conjunction with the methylesterase CheB in the chemosensory pathway. The point mutation in *cheR* of SV1 was also confirmed by sequencing the amplified PCR product using the primers listed in Supplementary Table 2. Furthermore, we deleted the *cheR* gene in the EPS+ host to verify the physiological functions of the *cheR* gene product (Figure 5B). As expected, a null *cheR* and *AT00_08765* mutant in which the chemosensory system was ineffective resulted in the smooth colony morphology (Figure 5A). For the SV2 variant strain, a single nucleotide deletion mutation in *bcsC* (*AT00_RS02190*) was located within the *bcs* (bacterial cellulose synthesis) operon, ranging from *AT00_RS02190* to *AT00_RS02225* as we have previously reported (Zeng et al., 2015). Similarly, the deletion mutation in *bcsC* of the SV2 variant strain was further confirmed by sequencing the PCR-amplification product. Intact *bcsC* is required for the expression of cellulose as reported in many microorganisms (Zogaj et al., 2001; Solano et al., 2002). Thus, it is hypothesized that *bcsC* mutation in the EPS+ strain results in the loss of cellulose production, leading to smooth colony morphology. Importantly, we previously demonstrated that deletion of the *bcs* gene within the cellulose operon in the *P. lipolytica* Δ*AT00_08765* host results in a morphological switch from wrinkled to smooth, suggesting that the cellulose operon and its product are required for the generation of wrinkled colony morphology (Zeng et al., 2015). Moreover, deletion of *cheR* or *bcs* genes in EPS+ host significantly reduced the cellulose production, and formed relatively less or rarely pellicle (Figure 5A). Since EPS+ derived smooth variant exhibited wild-type smooth colony morphology, we originally hypothesized that the smooth variants may recover the wild-type motility activity. However, results showed loss of *cheR* or *bcs* in EPS+ host only slightly increased swimming motility compared to that of the EPS+ strain (Figure 5C), suggesting *AT00_08765* and *cheR* involved in motility are independently from their role in controlling cellulose production. Moreover, the EPS+ strain harbors an intact single polar flagellar (Figure 5D), indicating that the reduced swimming motility of the EPS+ strain compared to that of the wild-type strain is independently from the biosynthesis of flagellar. Next, we deleted *cheR* in Δ*flhA* host to check whether *cheR* involved in the *flhA*-mediated wrinkled colony morphology. As we observed that Δ*flhA*Δ*cheR* double mutant strain developed a relative reduced wrinkled colony phenotype compared to that of the Δ*flhA* strain (Figure 5E), suggesting the AT00_08765-CheR signaling pathway may converge with the *flhA* dependent pathway in terms of the emergence of rugosity. Taken together, we demonstrated that the chemosensory pathway is crucial for the determination of wrinkled colony morphology by controlling the production of cellulose in *P. lipolytica*.

### Summary of the Genetic and Phenotypic Variations in *Pseudoalteromonas lipolytica* Biofilms

**FIGURE 6:**
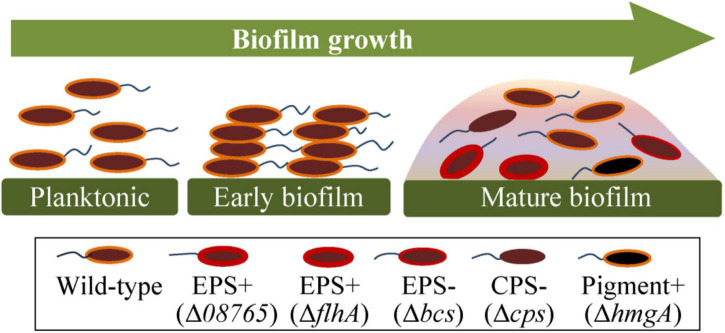
Summary of the genetic and phenotypic variations in *P. lipolytica* biofilms. During the growth of biofilms, the bacteria swim and follow an attachment to a supporting surface. The generated spatially heterogeneous microenvironment was supposed to help give rise to different types of genetic variants, such as EPS+ (increased polysaccharide production with reduced swimming motility), EPS– (reduced polysaccharide production), CPS– (reduced capsular polysaccharide production) and pigment production variants (increased pyomelanin production).

To gain a better insight into the evolution of genetic variants in *P. lipolytica* biofilms, we summarized the emergence of different types of genetic variants during the biofilm growth of *P. lipolytica*, each of which exhibited a specific phenotype (Figure 6). As noted thus far, the biofilm growth of *P. lipolytica* produced various phenotypic variants by introducing genome mutations in specific targets, such as *wspF-like* or *flhA* in wrinkled variants (EPS+) with higher cellulose production, *bcs* in smooth variants (EPS−) with lower cellulose production, in translucent variants (CPS−) with lower capsular polysaccharide production (Zeng et al., 2019) and *hmgA* in pigment production variants with higher pyomelanin production (Zeng et al., 2017). Although all of these different genetic variants can be produced during biofilm formation, it is noted that the emergence frequency of those different variants with specific phenotype mainly depends on the culture conditions (i.e., colony biofilm or pellicle biofilm, 2216E medium or HSLB medium, 25°C or 30°C). In general, the EPS+ and CPS− variants were generated at a relatively high frequency in *P. lipolytica* biofilm under laboratory culture condition. In all, the study of the molecular basis of these biofilm variants provides insights into the evolution within biofilms of *P. lipolytica*.

## Discussion

Previous studies showed that wrinkled colony morphology variants of *Pseudoalteromonas lipolytica* play important roles in marine antifouling and anticorrosion (Zeng et al., 2015; Liu et al., 2018). In this study, we identified that the emergence of wrinkled variants of *P. lipolytica* mainly arose due to mutations in the *AT00_08765* gene. Furthermore, we identified that the spontaneous mutation in *flhA* generated in *P. lipolytica* biofilms also caused wrinkled colony morphology, which is associated with cellulose overproduction. Moreover, a wrinkled variant harboring a mutation in *dgcB*, encoding a diguanylate cyclase, exhibited increased swimming motility. In contrast, DgcB overproduction decreased swimming motility while increasing cellulose production. In addition, by screening the suppressor of wrinkled variants during the biofilm growth of *P. lipolytica* EPS+, we found that the occurrence of spontaneous mutations in *cheR* or *bcsC* directly abolished the wrinkled phenotype in the *AT00_08765* variant strain. Thus, this study provides insights into a better understanding of genetic variation in marine *P. lipolytica* biofilms at the molecular level.

The emergence of wrinkled variants is commonly found during the development of bacterial biofilms among different species. In *P. aeruginosa*, wrinkled variants isolated from cystic fibrosis patients or laboratory biofilm cultures almost arose by introducing the mutations in *wspF* or *fleQ* (Smith et al., 2006; Mena et al., 2008; Gloag et al., 2019). In this study, a majority of wrinkled variants of *P. lipolytica* were also produced due to spontaneous mutations in *AT00_08765*, a *wspF-like* gene, suggesting that the chemosensory pathway remains a hot mutation target during the development of *P. lipolytica* biofilms. In *P. aeruginosa* PAO1, there are four chemosensory pathways that are encoded by five gene clusters. Among them, cluster III encodes the Wsp chemosensory system that comprises WspABCDEF, which regulates biofilm formation by stimulating c-di-GMP synthesis *via* the signaling protein WspR that is a diguanylate cyclase (Ortega et al., 2017). Although the amino acid sequences of AT00_08765 share 31% similarity with the WspF in PAO1, there is no gene near AT00_08765 with a product that resembles WspABCDE and WspR (Supplementary Table 4). Instead, AT00_08765 locates within a predicted chemosensory pathway (AT00_08740 to AT00_08780) that share highly similarity with the chemosensory pathway cluster I (PA1456 to PA1464) in PAO1 (Supplementary Table 5), which is essential for chemotaxis, controlling flagella-mediated motility (Ortega et al., 2017). Thus, *P. lipolytica* may experience quite a different signaling mechanism, rather than the canonical Wsp signaling pathway, to regulate cellulose production and swimming motility. Moreover, there is another WspF-like homolog present in the genome of *P. lipolytica*, AT00_17930 (Supplementary Table 4), which is more similar in size (343 aa) to WspF (335 aa), than AT00_08765 (378 aa), but with a lower percent-identity (29.8% for 17930 vs. 31.9% for 08765). It still remains to be determined whether mutation in *AT00_17930* would lead to the similar wrinkled phenotype. Further efforts are also needed to check the involvement of c-di-GMP and to explore the molecular signaling mechanism regarding on the emergence of wrinkled colony morphology in *P. lipolytica*.

The *P. aeruginosa* Wsp chemosensory consists of seven chemotaxis proteins comprised homologs of MCP (WspA), CheR (WspC), CheB (WspF), two CheWs (WspD and WspB), CheA–CheY hybrid (WspE), and a response regulator WspR (He and Bauer, 2014). A null *wspF* mutant, in which WspA is permanently methylated to activate signal transduction, exhibits elevated intracellular c-di-GMP production and further stimulates the production of Pel and Psl polysaccharides to exhibit the wrinkled colony morphology (Hickman et al., 2005). WspF (CheB-like) and WspC (CheR-like) constitute a feedback mechanism that constantly controls the state of WspA between methylation and demethylation, which allows the regulation of signal transduction (He and Bauer, 2014). As manifested in this study, we found that a *AT00_08765* (*cheB*) mutant restores the wild-type smooth phenotype by introducing a point mutation in *AT00_09010* (*cheR*), which may abolishes the signal transduction. FleQ, a c-di-GMP binding protein, directly represses the transcription of the polysaccharide biosynthesis operon, while activating the transcription of many flagellar genes, such as *flhA*, *fliE* and *fliL*. Loss of *fleQ* relieves the repression of the polysaccharide biosynthesis operon and thus leads to wrinkled colony morphology (Baraquet and Harwood, 2016). In fact, the FleQ-like protein (AT00_08895) in *P. lipolytica* shares 51% similarity (99% coverage) with that of *P. aeruginosa*. We also constructed a *AT00_08895* deletion mutant in *P. lipolytica*, which leds to wrinkled colony morphology (Supplementary Figure 2). However, we have not identified wrinkled variants of *P. lipolytica* that harbor spontaneous mutations in *AT00_08895* in this study.

Genetic screening of the genes essential for the wrinkled colony phenotype identified many genes required for polysaccharide biosynthesis, such as *bcs* in *P. fluorescens* (Lind et al., 2017) and *E. coli* (Serra et al., 2013), *pel* and *psl* in *P. aeruginosa* (Friedman and Kolter, 2004) and *vps* in *V. cholera* (Rashid et al., 2003). Additionally, *V. cholerae* harbors mutations in flagellar genes, such as *flrABC*, *flhAB*, and *fliGMN*, also displays a wrinkled colony morphology that is correlated with VPS production, suggesting that the absence of the flagella structure is an important signal to increase exopolysaccharide synthesis (Watnick et al., 2001; Wu et al., 2020). In this study, we identified that the spontaneous mutation in *flhA* causes a non-motile and wrinkled colony phenotype in *P. lipolytica* which is linked to the cellulose production. Although spontaneous mutation in *flhA* are rarely found in other species, other genes, such as *flhDC* and *fleQ*, which play important roles in controlling swimming motility, are hot mutation targets in many species under adverse conditions (Mena et al., 2008; Wang and Wood, 2011), suggesting that swimming motility is one of the most highly regulated phenotypes in bacteria. *flhA* encodes a conserved membrane component of the flagellar type III protein secretion system (T3SS), which controls the export of hook protein, flagellin, as well as other non-flagellar proteins. FlhA also acts as a master regulator that regulates transcription in the RpoN (σ54 sigma factor) and FliA (σ28 sigma factor) regulons, such as GGDEF related genes for some bacteria (Niehus et al., 2004; Yang et al., 2009). Thus, it is likely that loss of *flhA* could stimulate the production of c-di-GMP through a complicated regulatory network and then promote the production of exopolysaccharides. Further studies should be focused on the molecular mechanism related to *flhA*-mediated signal transduction in *P. lipolytica*.

In this study, the molecular basis of the wrinkled phenotype for the V4 variant strain remains unknown, but is hypothesized to be due to the undetected mutation site caused by IS jumping or genomic rearrangements. We previously reported that IS*5* actively inserted in the *cps* locus at a higher frequency during the growth of biofilms in *P. lipolytica* (Zeng et al., 2019). Thus, it remains to be determined whether IS jumping at a specific gene is responsible for the generation of wrinkled phenotype in the V4 variant. Within the V4 variant, we found a mutation in *AT00_RS12195* (*dgcB*), encoding a DGC protein that is capable of synthesizing the c-di-GMP, which is associated with increased swimming motility compared to other wrinkled variants. Although deletion of *AT00_RS12195* in the wild-type strain imposed a minor effect on swimming motility and cellulose production, expression of AT00_RS12195 through a pBBR1MCS-based plasmid significantly decreased swimming motility while increasing cellulose production, suggesting that *AT00_RS12195* still plays an important role in the c-di-GMP-mediated signaling process. In fact, greater than 40 putative DGCs were found resided within the genome of *P. lipolytica*. A wrinkled variant thus might stimulate the expression of more than one DGC, each in principle capable of inhibiting swimming motility. Hence, wrinkled variant loss of one of these DGC targets, such as AT00_RS12195, might increase swimming motility to a certain extent.

Experimental evolution is increasingly being used to study microbial evolutionary processes, providing valuable information not only for expanding our understanding of bacterial evolution but also for exploring helpful microorganisms with beneficial traits (McDonald, 2019). We previously demonstrated that *P. lipolytica* biofilm produced different genetic variants with different phenotypic traits, such as pigmented, translucent and wrinkled colony morphology, all of which play important roles in antifouling or anticorrosion activity (Zeng et al., 2015, 2017, 2019; Liu et al., 2018). A great advantage of experimental evolution is that it is driven by natural selection, so the fitness acquired by spontaneous mutation in different variant strains might confer a more resilient phenotype to adapt to unfavorable environments. Thus, the elucidation of the molecular basis for these biofilm variants might provide valuable information for further constructing beneficial engineering strains with greater antifouling and anticorrosion activities.

## Data Availability Statement

The datasets presented in this study can be found in online repositories. The names of the repository/repositories and accession number(s) can be found in the article/Supplementary Material.

## Author Contributions

ZZ and YG developed the concept of this study and are main contributors to writing the manuscript. ZZ, SL, and WW performed all experiments, carried out the data analysis, and prepared the figures. QL, YW, TX, and YG contributed to the manuscript edit and review. All authors read and approved the final manuscript.

## Conflict of Interest

The authors declare that the research was conducted in the absence of any commercial or financial relationships that could be construed as a potential conflict of interest.

## Publisher’s Note

All claims expressed in this article are solely those of the authors and do not necessarily represent those of their affiliated organizations, or those of the publisher, the editors and the reviewers. Any product that may be evaluated in this article, or claim that may be made by its manufacturer, is not guaranteed or endorsed by the publisher.
