# Supplementary material for: Molecular Basis of Wrinkled Variants Isolated From Pseudoalteromonas lipolytica Biofilms
Source: Front Microbiol. 2022 Feb 28;13:797197. doi: 10.3389/fmicb.2022.797197 (PMC8919034; doi:10.3389/fmicb.2022.797197)
Supplement: Supplementary file 1 [file Data_Sheet_1.docx]

**Table S1.** Bacterial strains and plasmids used in this study.

| Strains and plasmids | Relevant characteristics | Source |
| --- | --- | --- |
| Strains |  |  |
| *E.coli* WM3064 | RP4(tra) in chromosome, DAP- | (Dehio and Meyer 1997) |
| *Pseudoalteromonas lipolytica* SCSIO_04301 | Wild-type strain | (Zeng, et al 2014) |
| *P. lipolytica* wrinkled variants (V1 to V24) | Wrinkled colony morphology | This study |
| *P. lipolytica* EPS+ | point mutation in *AT00_08765* | (Zeng, et al 2018) |
| EPS+ smooth variants  (SV1 and SV2) | Smooth colony morphology | This study |
| *P. lipolytica* Δ*flhA* | *flhA* gene deletion | This study |
| *P. lipolytica* Δ*dgcB* | *dgcB* gene deletion | This study |
| *P. lipolytica* EPS+ Δ*cheR* | *cheR* gene deletion in EPS+ host | This study |
| *P. lipolytica* EPS+ Δ*bcs* | *bcsZB* gene deletion in EPS+ host | This study |
| *P. lipolytica* EPS+ Δ*dgcB* | *dgcB* gene deletion in EPS+ host | This study |
| *P. lipolytica* Δ*flhA*Δ*cheR* | *flhA* and *cheR* genes deletion | This study |
| *P. lipolytica* Δ*fleQ* | *fleQ* gene deletion | This study |
| Plasmids |  |  |
| pK18mob*sacB*-ery | Gene knockout vector, Kan^r^, Ery^r^ | (Wang et al. 2015) |
| pK18mob*sacB*-ery- *flhA* | Vector for deleting *flhA* | This study |
| pK18mob*sac*B-ery-*dgcB* | Vector for deleting *dgcB* | This study |
| pK18mob*sacB*-ery-*cheR* | Vector for deleting *cheR* | This study |
| pK18mob*sacB*-ery-*fleQ* | Vector for deleting *fleQ* | This study |
| pBBR1MCS-Cm | Expression vector, Cm^r^ | (Wang et al. 2015) |
| pBBR1MCS- *AT00_08765* | Vector for expression *AT00_08765* | (Zeng et al. 2015) |
| pBBR1MCS-*flhA* | Vector for expression *flhA* | This study |
| pBBR1MCS-*flhA* (variant) | Vector for expression variant *flhA* | This study |
| pBBR1MCS-*dgcB* | Vector for expression *dgcB* | This study |
| pBBR1MCS- *dgcB* (variant) | Vector for expression variant *dgcB* | This study |

**Table S2.** Sequences of primers used in this study.

| **Primer name** | **Sequence** |
| --- | --- |
| **In-frame deletions** |  |
| cheR-up-S | acgacggccagtgccaagcttTTGTTCCGGCTCTGGTTGTT |
| cheR-up-A | gtgcGATCGTATTCGCTTTGTTGTAAGTG |
| cheR-down-S | caaagcgaatacgatcGCACTTAATCCAAAAGGCTACCTG |
| cheR-dwon-A | agtcactggggatcctctagaGGTGATTCGGGTTGTATTCTATTTG |
| cheR-SF | CGGTGTACAAACTGAACTCGCTA |
| cheR-SR | TAGGTTGCTAAGGAAAGATTACTTTTTC |
| cheR-LF | GATTTGTTGAAGTTGCTCGT |
| cheR-LR | TTGGCGTGTTTGTCTCATTC |
| flhA-up-S | acgacggccagtgccaagcttGCCACAAGCATCACAAAGAGC |
| flhA-up-A | gacataccaacCCAACAGCATAGTTACCGCCG |
| flhA-down-S | tgctgttggGTTGGTATGTCATCTGAAATCCCTG |
| flhA-down-A | agtcactggggatcctctagaAATCTAAATCGTCGAAGCCTGTG |
| flhA-SF | GGTTATTTTACCTCTGCCTCC |
| flhA-SR | GACAATTTCAACACCATTCGCT |
| flhA-LF | ATTGAAGTTTGCGGTGGTTG |
| flhA-LR | TAAAACACGCCATTTGCTCC |
| dgcB-up-S | acgacggccagtgccaagcttTTCCATACCCGAAACAGAGCG |
| dgcB-up-A | gAATAAAGGGGGCTAGTAGGTTAAAAT |
| dgcB-down-S | cctactagccccctttattCACTTGAAAAAGCCGTAGCACC |
| dgcB-down-A | agtcactggggatcctctagaTCGATGTCAGGCAGTATAACCAGC |
| dgcB-SF | GCATCGAAAAAACGTACCCCA |
| dgcB-SR | AAGCCTAACTCGTAACCCACC |
| dgcB-LF | TACCGTCGCCCCAAATAACC |
| dgcB-LR | CGCAGAACGTACAAAAGCCTC |
| fleQ-up-S | agtcactggggatcctctagaTGATTTTGGTTTTATCTCCGAAGA |
| fleQ-up-A | gtcataacgctcCTCCGATAAAAGTGAGGGCTGC |
| fleQ-down-S | tatcggagGAGCGTTATGACTATGTTGTAGCGC |
| fleQ-down-A | gccagtgccaagcttgcatgcCTCACCCACATACTCTTCTTGCTG |
| fleQ-SF | ACTATTTTTTGGCGTTGATG |
| fleQ-SR | AATGGCAGTTCGTTGGGG |
| fleQ-LF | AAGCTTTACAAACCCCACT |
| fleQ-LR | AATACCACAACCCCCGCA |
| **Complementation** |  |
| flhA-pBBR-F | gtgaccgtgtgcttcgaattcAACCCGACTCACTATTCGGTTG |
| flhA-pBBR-R | gtcgacggtatcgataagcttATTTTCATGGTACAACCCCTTTACT |
| dgcB-pBBR-F | gtgaccgtgtgcttcgaattcCATCGAAAAAACGTACCCCATT |
| dgcB-pBBR-R | gtcgacggtatcgataagcttACATAAGCACTCTCCTAGCTCGTCA |
| **Sequencing primers** |  |
| AT00_08765-f | TTTACTGCAAGGTACACCA |
| AT00_08765-r | TGAACAAAGCACTGACAAC |
| cheR-f | CGGTGTACAAACTGAACTCGCTA |
| cheR-r | TAGGTTGCTAAGGAAAGATTACTTTTTC |
| bcsC-f | ACACCGTGCTGTTTTACCG |
| bcsC-r | TTGGCGGTATGCTTCTTGT |
| flhA-f | TTGAGTTTGGTATGTTTCCG |
| flhA-r | TTCATCTTTCTGGTCTGGC |
| dgcB-f | CATCGAAAAAACGTACCCCATT |
| dgcB-r | ACATAAGCACTCTCCTAGCTCGTCA |
| pK18-f | ATTCCGCTGGCAGCTTAAG |
| pK18-r | GGTAACGCCAGGGTTTTCC |
| pBBR1MCS-f | TCGTTAAATAGCCGCTTATG |
| pBBR1MCS-r | AATTTCACACAGGAAACAGC |

**Table S3.** Statistics of the whole-genome re-sequencing data.

| Samples | Reads length  (nt) | Raw data (Mb) | Total reads | Clean data (Mb) | N  (ppm) | GC (%) | Genome coverage (%) | Sequencing depth (X) |
| --- | --- | --- | --- | --- | --- | --- | --- | --- |
| SV1 | 150 | 3515 | 23197190 | 3450 | 8.81 | 41.44 | 99.83% | 547 |
| SV2 | 150 | 3827 | 25252310 | 3754 | 9.15 | 41.48 | 99.83% | 593 |
| V3 | 150 | 3384 | 22316100 | 3318 | 9.03 | 41.43 | 99.83% | 526 |
| V4 | 150 | 3728 | 21792872 | 3659 | 8.9 | 41.45 | 99.83% | 575 |

Note: N indicates the number of N per one million bases. Genome coverage was calculated by using the genome of *Pseudoalteromonas lipolytica* (GenBank assembly accession: GCA_000576675.1) as reference.

**Table S4.** The homolog of the *wsp*-like system in *Pseudoalteromonas lipolytica* compared to the *wsp* system in *Pseudomonas aeruginosa* strain PAO1.

| Genes in PAO1 | Homolog genes in SCSIO 04301 | Coverage in amino acid sequences | Identity | Genes annotation in SCSIO 04301 |
| --- | --- | --- | --- | --- |
| *wspA* | AT00_13635 | 100% | 26% | methyl-accepting chemotaxis protein |
| *wspB* | AT00_17950 | 57% | 29% | chemotaxis protein CheW |
| *wspC* | AT00_09010 | 45% | 30% | chemotaxis methyltransferase CheR |
| *wspD* | No found |  |  |  |
| *wspE* | AT00_17955 | 48% | 31% | chemotaxis sensor kinase CheA |
| *wspF* | AT00_17930 | 98% | 30% | chemotaxisresponse regulator CheB |
|  | AT00_08765 | 98% | 31% | chemotaxis-specific methylesterase |
| *wspR* | AT00_00325 | 90% | 36% | Diguanylate cyclase PleD |

**Table S5**. Chemosensory system (AT00_08740- AT00_08780) in *Pseudoalteromonas lipolytica* was predicted by comparing those of which in *Pseudomonas aeruginosa* strain PAO1.

| Genes in SCSIO 04301 | Homolog genes in PAO1 | Gene name | Protein sequence  coverage | Protein sequence  identity | Genes annotation in PAO1 |
| --- | --- | --- | --- | --- | --- |
| AT00_08740 | PA1464 | cheW | 96% | 73% | purine-binding chemotaxis protein |
| AT00_08745 | PA1463 | cheW | 57% | 45% | Hypothetical protein |
| AT00_08750 | PA1462 | parA | 99% | 50% | plasmid partitioning protein |
| AT00_08755 | PA1461 | motB | 83% | 37% | flagellar motor MotD |
| AT00_08760 | PA1460 | motA | 96% | 54% | flagellar motor MotC |
| AT00_08765 | PA1459 | cheB | 98% | 58% | chemotaxis-specific methylesterase |
| AT00_08770 | PA1458 | cheA | 99% | 57% | chemotaxis family, sensor kinase |
| AT00_08775 | PA1457 | cheZ | 95% | 44% | protein phosphatase CheZ |
| AT00_08780 | PA1456 | cheY | 99% | 80% | chemotaxis protein CheY |

**Figure S1.** Cellulose production was examined and measured using Congo red for the P. lipolytica wild-type, EPS+ and 24 wrinkled variants.

**
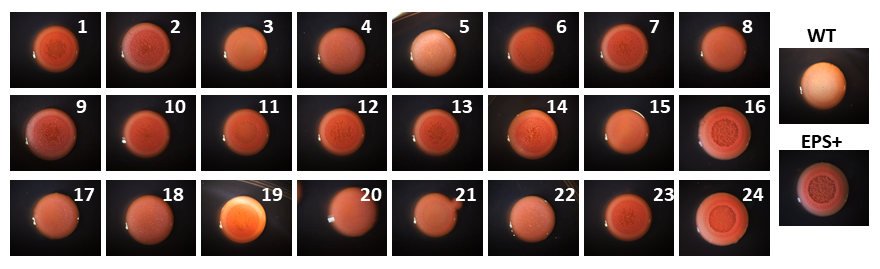
**

**Figure S2.** Deletion of the *fleQ*-like gene (AT00_08895) leads to a wrinkled colony morphology in *P. lipolytica*.

(A) In-frame deletion of *AT00_08895* in *P. lipolytica* wild-type strain was confirmed by PCR using four primer sets (LF&LR, SF&SR, LF&SR and SF&LR) flanking the open reading frame of *AT00_08895* (up panel). M indicated marker. Lanes 1, 3, 5 and 7 used DNA from the wild-type strain, and lanes 2, 4, 6 and 8 used DNA from the *AT00_08895* mutant strain. (B) The colony morphologies of the wild type and deletion mutant strains were examined by stereoscopic microscopy after three days of incubation on SWLB agar plates.

**
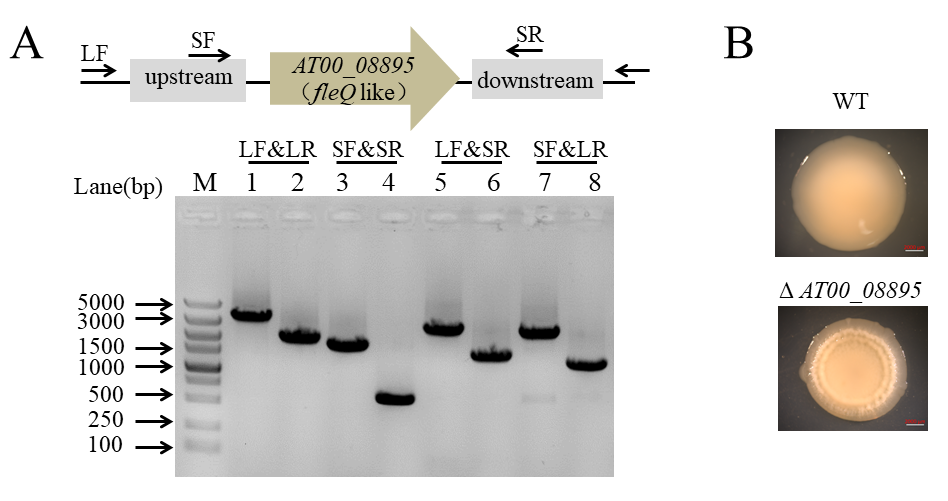
**

**Reference**

Dehio C, Meyer M. (1997). Maintenance of broad-host-range incompatibility group P and group Q plasmids and transposition of Tn*5* in Bartonella henselae following conjugal plasmid transfer from *Escherichia coli*. *J Bacteriol* 179**,** 538-540.

Liu, T., Guo, Z.W., Zeng, Z.S., Guo, N., Lei, Y.H., Liu, T., et al. (2018). Marine bacteria provide lasting anticorrosion activity for steel via biofilm-induced mineralization. *ACS Appl Mater Inter* 10(46)**,** 40317-40327.

Wang, P., Yu, Z., Li, B., Cai, X., Zeng, Z., Chen, X., et al. (2015). Development of an efficient conjugation-based genetic manipulation system for *Pseudoalteromonas*. *Microb Cell Fact* 14**,** 11.

Zeng Z, Dai S, Xie Y, Tian X, Li J, Wang X (2014) Genome sequences of two *Pseudoalteromonas* strains isolated from the South China Sea. Genome A 2: e00305-14.

Zeng, Z.S., Guo, X.P., Li, B.Y., Wang, P.X., Cai, X.S., Tian, X.P., et al. (2015). Characterization of self-generated variants in *Pseudoalteromonas lipolytica* biofilm with increased antifouling activities. *Appl Microbiol Biot* 99(23)**,** 10127-10139.
